# Comparative analyses of caste, sex, and developmental stage‐specific transcriptomes in two *Temnothorax* ants

**DOI:** 10.1002/ece3.6187

**Published:** 2020-03-30

**Authors:** Claudia Gstöttl, Marah Stoldt, Evelien Jongepier, Erich Bornberg‐Bauer, Barbara Feldmeyer, Jürgen Heinze, Susanne Foitzik

**Affiliations:** ^1^ Zoology/Evolutionary Biology University of Regensburg Regensburg Germany; ^2^ Institute of Molecular and Organismic Evolution Johannes Gutenberg University Mainz Germany; ^3^ Evolutionary Bioinformatics Institute for Evolution and Biodiversity Westfälische Wilhelms University Münster Germany; ^4^ Senckenberg Biodiversity and Climate Research Centre Frankfurt am Main Germany

**Keywords:** caste, developmental stages, gene expression, slave‐making ants, transcriptomics

## Abstract

Social insects dominate arthropod communities worldwide due to cooperation and division of labor in their societies. This, however, makes them vulnerable to exploitation by social parasites, such as slave‐making ants. Slave‐making ant workers pillage brood from neighboring nests of related host ant species. After emergence, host workers take over all nonreproductive colony tasks, whereas slavemakers have lost the ability to care for themselves and their offspring. Here, we compared transcriptomes of different developmental stages (larvae, pupae, and adults), castes (queens and workers), and sexes of two related ant species, the slavemaker *Temnothorax americanus* and its host *Temnothorax longispinosus.* Our aim was to investigate commonalities and differences in group‐specific transcriptomes, whereupon across‐species differences possibly can be explained by their divergent lifestyles. Larvae and pupae showed the highest similarity between the two species and upregulated genes with enriched functions of translation and chitin metabolism, respectively. Workers commonly upregulated oxidation‐reduction genes, possibly indicative of their active lifestyle. Host workers, but not workers of the slavemaker, upregulated a “social behavior” gene. In slavemaker queens and workers, genes associated with the regulation of transposable elements were upregulated. Queens of both species showed transcriptomic signals of anti‐aging mechanisms, with hosts upregulating various DNA repair pathways and slavemaker queens investing in trehalose metabolism. The transcriptomes of males showed enriched functions for quite general terms realized in different genes and pathways in each species. In summary, the strong interspecific commonalities in larvae, pupae, and workers were reflected in the same enriched Gene Ontology (GO) terms. Less commonalities occurred in the transcriptomes of queens and males, which apparently utilize different pathways to achieve a long life and sperm production, respectively. We found that all analyzed groups in this study show characteristic GO terms, with similar patterns in both species.

## Graphical abstract

The aim of the present study is to identify characteristic gene expression patterns of ant larvae, worker pupae, workers, queens, and males. The study uncovers commonalities and differences within and between the two ant species *Temnothorax americanus* and *Temnothorax longispinosus*—a parasitic slave‐making ant species and its host.

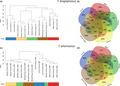

## 1 INTRODUCTION

The ecological success of social Hymenoptera is based on tight cooperation and an efficient division of labor in their colonies. Reproduction is monopolized by one or a few female individuals (the queens), whereas all other colony members (the workers) take care of the brood and the queen, build and defend the nest, and forage for food (Hölldobler & Wilson, 1990), but never reproduce. The altruistic behavior of workers can be exploited by social parasites, such as slave‐making ants.

Freshly mated ant queens typically found new colonies independently or may be re‐adopted by their natal colony. In contrast, young slavemaker queens usurp a nest of another, closely related ant species (“Emery's rule,” Emery, 1909). They have to drive away or kill the resident queen and, in many species, all adult workers. Host workers emerging from the conquered brood take care of the slavemaker's own offspring (Buschinger, 1986; d'Ettorre & Heinze, 2001). Slavemaker workers have lost the ability to nurse the brood and to forage and thus rely entirely on the help of their enslaved workforce. To replenish or increase this workforce, they conduct raids on host colonies to steal brood. Young slave‐making workers also participate in reproduction and lay unfertilized male‐destined eggs even in the presence of the queen (Brunner, Trindl, Falk, Heinze, & d'Ettorre, 2005; Foitzik & Herbers, 2001; Franks & Scovell, 1983; Heinze, 1996), while host workers typically reproduce only in the queens' absence (Bourke, 1988). As fertility is often positively associated with longevity (Kuhn & Korb, 2016) and egg‐laying queens and workers live longer than their nonreproductive nestmates (Heinze & Schrempf, 2012; Kohlmeier et al., 2017; Tsuji, Peeters, & Hölldobler, 1998), workers of slave‐making ants might be more long‐lived than host workers. Hence, though typically closely related, slave‐making ants and their hosts differ substantially in their life histories.

The morphological and life history differences between slavemakers and their hosts are likely reflected in different patterns of gene expression. Here, we compare gene expression of different castes, developmental stages, and sexes of the slave‐making ant, *Temnothorax americanus* (Emery, 1895) (formerly *Protomognathus americanus*) and its primary host *Temnothorax longispinosus* (Roger, 1863). The aim of our study is to determine the phenotype‐specific expression patterns between castes and sexes and to compare them between species showing different lifestyles—slavemaker and host. Within the ant subfamily Myrmicinae, the *Formicoxenus*‐species group (sensu Blaimer, Ward, Schultz, Fisher, & Brady, 2018) is a hot spot for the evolution of slave‐making, with at least four independent origins within the genus *Temnothorax* alone (Alleman, Feldmeyer, & Foitzik, 2018; Beibl, Stuart, Heinze, & Foitzik, 2005). Our two focal species have been well‐studied as a model for an ongoing coevolutionary arms race between social parasites and hosts (e.g., Foitzik, DeHeer, Hunjan, & Herbers, 2001; Jongepier, Kleeberg, Job, & Foitzik, 2014, Kaur et al., 2019). For example, analyses of brain gene expression in *T. americanus* and *T. longispinosus* workers during scouting and tandem running revealed a divergent molecular regulation of seemingly similar behavior in the two related species (Alleman, Stoldt, Feldmeyer, & Foitzik, 2019).

The aim of the present study is to identify characteristic expression patterns of larvae, worker pupae, workers, queens, and males and to search for commonalities and differences between the two ant species *T. americanus* and *T. longispinosus*. On the one hand, we contrast here whole‐body gene expression of ants with divergent morphological and behavioral phenotypes, so that the transcriptomic signatures should clearly differ between these groups within each species. On the other hand, larvae of both species are expected to overexpress growth‐related genes, and pupae should commonly overexpress genes associated with tissue reconstruction and cuticular synthesis (Dubrovsky, 2005). Queens of the two species are predicted to show strong transcriptomic correlates of reproduction and longevity (e.g., Negroni, Foitzik, & Feldmeyer, 2019). Due to their different life histories, worker and queen transcriptomes might show less interspecies commonalities in gene expression. The short‐lived males are expected to strongly invest in sperm production and less in anti‐aging mechanisms.

## 2 MATERIAL AND METHODS

### 2.1 Collection and colony maintenance

Colonies of the ant species *T. americanus* and *T. longispinosus* were collected in June 2016 at the E.N. Huyck Preserve, Rensselearville, New York, USA (42°31′43.8″N, 74°09′44.7″W), from which collection permits were obtained. After transfer to the laboratory, ant colonies were kept in three‐chambered nest boxes with a plaster floor and a nest site consisting of a Plexiglas® frame sandwiched between two microscope slides covered by red foil. The ants were maintained in a climate chamber at artificial summer conditions at 23°C with 12 hr day/12 hr night cycle until sampling in July and August 2016. The ants were fed with crickets and honey twice a week. Fresh water was available ad libitum.

### 2.2 Extraction, sequencing, and de novo assembly

RNA extraction of all samples was conducted in November 2016 using the RNeasy mini extraction Kit (Qiagen, Inc.). For each species, we analyzed a medium‐sized larva, an unpigmented worker pupa, a male, an adult worker, and a fertile queen from three different colonies each (*N* = 3). We extracted RNA from whole bodies, as we had no prior assumptions as to which tissue differed most strongly in gene expression between our groups. To determine the sex of the larvae, we tested heterozygosity. For *T. longispinosus,* the test shows clearly that all larvae are female. For *T. americanus,* the samples show a heterogeneous pattern of heterozygosity, for example, lowest heterozygosity in two of the queens; thus, we cannot clarify whether the larvae in this species are male or female.

Library construction and sequencing were conducted at BGI Hong Kong on an Illumina HiSeq 4000. For rRNA depletion, a poly‐A selection was conducted. With a read length of 100 bp, the libraries resulted in a total of 40 million paired‐end reads (4 GB) for each sample. The quality of raw reads was assessed using “FastQC” v0.11.4 (Andrews, 2010), and Illumina adapters were cut from all sequences using “Trimmomatic” v0.32 (Bolger, Lohse, & Usadel, 2014). We conducted a de novo transcriptome assembly for both species using “Trinity” v2.4.0 (Grabherr et al., 2011), including the paired forward and reverse sequences of the 15 samples for each species. For *T. longispinosus,* the transcriptome had a total length of 275,441,418 bp, equivalent to 62,775 Trinity “genes” or 160,971 Trinity transcripts with a GC content of 42.32%. The transcriptome of *T. americanus* had a total length of 267,319,768 bp, equivalent to 86,416 Trinity “genes” or 228,156 Trinity transcripts with a GC content of 42.45%. The reads of the samples mapped back to their corresponding transcriptomes with an average back mapping rate of 82.27% (for *T. longispinosus*) and 66.17% (for *T. americanus*).

### 2.3 Differential gene expression and enrichment analyses

To investigate the clustering of samples according to the expression profiles of the 20,000 most variant genes, we used “WGCNA” v1.64‐1 (Langfelder & Horvath, 2008), a package of the software “R” (R Core Team, 2018). Paired forward and reverse reads were mapped to the corresponding transcriptomes using “Bowtie2” v2.3.5 (Langmead & Salzberg, 2012), and read counts were estimated using “RSEM” v1.3.0 (Li & Dewey, 2011). To identify genes upregulated in a specific developmental stage or caste, we calculated the differentially expressed genes (DEGs) in a pairwise comparison within each species using the R package “DESeq2” v1.14.1 (Love, Anders, & Huber, 2014) as embedded in Bioconductor v3.4 (Gentleman et al., 2004). “DESeq2” models read counts assuming a negative binomial distribution using generalized linear models with logarithmic link to access the log2 fold change between two conditions of one factor or even designs including more than one factor. For our pairwise comparisons, we used the Wald test as implemented in “DESeq2” to obtain genes that differed significantly in their expression between two groups of the only factor in our design, developmental stage/caste. This was done for all possible 20 pairwise comparisons, only keeping genes with a positive log fold change and an FDR adjusted *p*‐value < .05. We then merged the lists of genes that were upregulated in the same developmental stage or caste compared to any of the other groups (see Supplement S1 and S2) and created a Venn diagram of the resulting five sets using the webtool provided on http://bioinformatics.psb.ugent.be/webtools/Venn. This allowed us to extract the genes that were only upregulated in a single developmental stage or caste, in the following referred to as “privately upregulated,” as well as the genes that were upregulated in all but one developmental stage/caste, which we here refer to as “privately downregulated” in the latter. To compare the number of DEGs across species, we had to take the transcriptome size into account, which was larger in *T. americanus*.

The nucleotide sequences of both transcriptomes were translated into amino acid sequences using “Transdecoder” v. 3.0.1 (https://github.com/TransDecoder). We classified these sequences into families and predicted domains by running “Interproscan” v. 5.27‐66.0 (Quevillon et al., 2005) on the amino acid sequences. For functional annotation, we performed a GO enrichment analyses with “TopGO” v. 2.28.0 (Alexa & Rahnenfuhrer, 2010) using Fisher's exact test to test for enrichment of GO terms in the sets of privately up‐ or downregulated genes compared to the whole transcriptome of the focal species. We only took terms into account, which had at least 10 annotations in the transcriptome (node size 10). We illustrated the GO terms in each gene set, which had a *p*‐value < .05, in a word cloud, adjusting the font size according to the *p*‐value (see Supplement S3 and S4)(font size adjustment = −1 × log (*p*‐value)) using the R package “tagcloud” v. 0.6 (Weiner, 2015).

### 2.4 Analysis of orthologous genes

First, we investigated whether the privately differentially expressed genes of the several groups show sequence similarity, that is, correspond to the same gene between species. Therefore, we ran “Orthofinder” v2.2.3 (Emms & Kelly, 2015) to find upregulated orthologous gene sequences. Only orthogroups with exactly one sequence per species were considered for downstream analyses (from now on referred to as single‐copy orthologs). These single‐copy orthologs were used to compare gene expression patterns between species for each developmental stage and caste.

Additionally, we performed a separate gene expression analysis by obtaining single‐copy orthologs between the two transcriptomes and afterwards again running DESeq2 including phenotype and species as well as their interaction in the model. Afterwards, we compared the expression of genes inside each caste between species again in pairwise comparisons. We then performed GO enrichment analyses on the resulting gene lists for the genes upregulated in *T. longispinosus* and the genes upregulated in *T. americanus* separately.

GO enrichment was again performed as described before, but since only the orthologous genes were used for the gene expression analysis, GO enrichment analysis was also only performed with the entirety of orthologous genes as “universe.” Each orthogroup was associated with two sequences, one in *T. americanus* and one in *T. longispinosus*. Although the sequences in each orthogroup should be very similar and therefore yield the same GO annotations, there were some minor differences in the annotations of the orthologs for both species. Therefore, the annotated GO terms were merged so that in case of a conflict both the GO terms of *T. americanus* as well as the ones of *T. longispinosus* were used for this gene as “universal” annotations.

## 3 RESULTS

### 3.1 Clustering of transcriptomes

**Figure 1:**
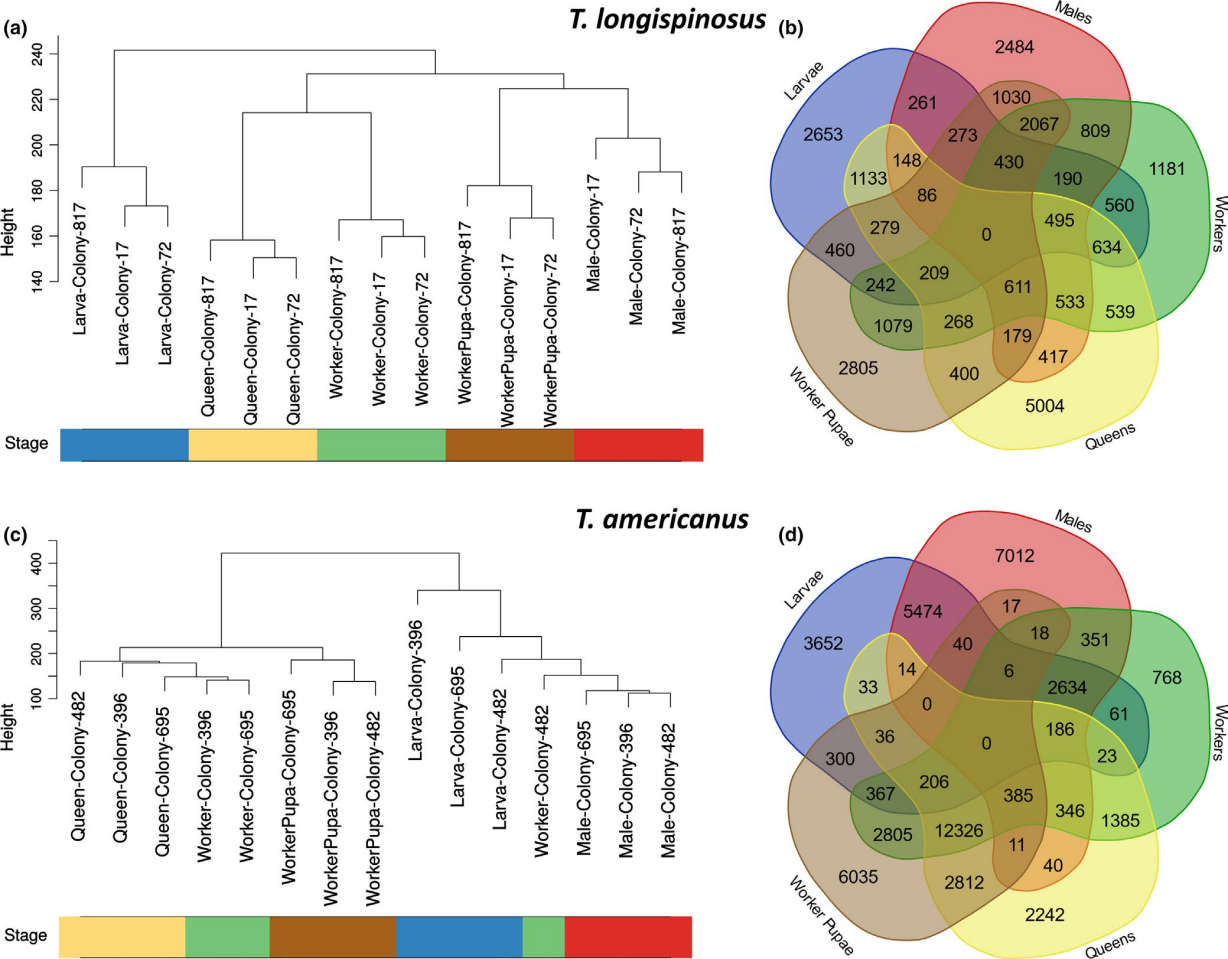
Gene expression differences between groups of individuals in the slave‐making ant *Temnothorax americanus* and its main host *Temnothorax longispinosus.* Dendrograms were created using UPGMA with the Euclidean distance between all genes of the samples as input for (a) *T. longispinosus* and (c) *T. americanus*. Venn diagrams show the number of genes overexpressed with an adjusted *p*‐value < .05 in each phenotype and the overlap between these calculated using Wald test pairwise comparisons as implemented in DESeq2 for (b) *T. longispinosus* and (d) *T. americanus*

Samples of the host species *T. longispinosus* cluster according to developmental stage, caste, and sex, with larvae being the outgroup to all others (Figure 1a). The queen and worker groups are grouped together, and so are the males and worker pupae. Clustering was much less clear in the slavemaker *T. americanus.* Here, only the worker pupae and males clustered in one group each. Instead, samples of *T. americanus* were grouped into two major branches, one containing the worker pupae, workers, and queens, the other all males, larvae, and one odd worker (Figure 1b). Removing the latter worker from the analysis did not change the allocation of the other specimens. The separation of the two major branches was deeper than in *T. longispinosus*, suggesting stronger differences between males and larvae compared to all the other samples of *T. americanus*.

### 3.2 Gene expression analysis

In our within‐species analyses, we found a total of 27,459 (17.06%) unique genes to be upregulated in at least one caste of *T. longispinosus*. The largest number of privately upregulated genes was found in queens (5,004, i.e., 3.1% of all transcripts), while workers had the lowest number of privately upregulated genes (1,181, 0.7%). Queens and workers co‐upregulated 3,289 genes (2.0%), while worker pupae and larvae shared only 1,979 (1.2%) of the upregulated genes.

In *T. americanus,* a total of 49,585 (21.73%) genes were upregulated in at least one caste. Again, workers exhibited the lowest number of private DEGs (768, 0.3%), which was significantly less than in *T. longispinosus* (*χ* ^2^ = 297.78, *df* = 1, *p* < .00001). *Temnothorax americanus* queens had less than 1.0% of uniquely upregulated genes (2,242), that is, significantly less than the DEGs detected in host queens (*χ* ^2^ = 2,333.2, *df* = 1, *p* < .00001). The highest overlap of upregulated genes occurred among all female specimens, that is, workers, worker pupae, and queens, which commonly upregulated 12,326 genes (5.4%). This is a much higher overlap than that between the respective castes in *T. longispinosus* (268 genes (0.17%); *χ* ^2^ = 8,260.5, *df* = 1, *p* < .00001).

Besides other differences in the number of upregulated genes in the different groups between species, we found more DEGs among developmental stages, castes, and sexes in *T. americanus* (including shared genes) than in *T. longispinosus* (*χ* ^2^ = 3,786.9, *df* = 1, *p* < .00001).

The analysis of the downregulated genes gave no further information about group‐specific functions as the resulting terms were very general. Therefore, this study concentrates on the upregulated genes only.

### 3.3 Enrichment analyses

**Figure 2:**
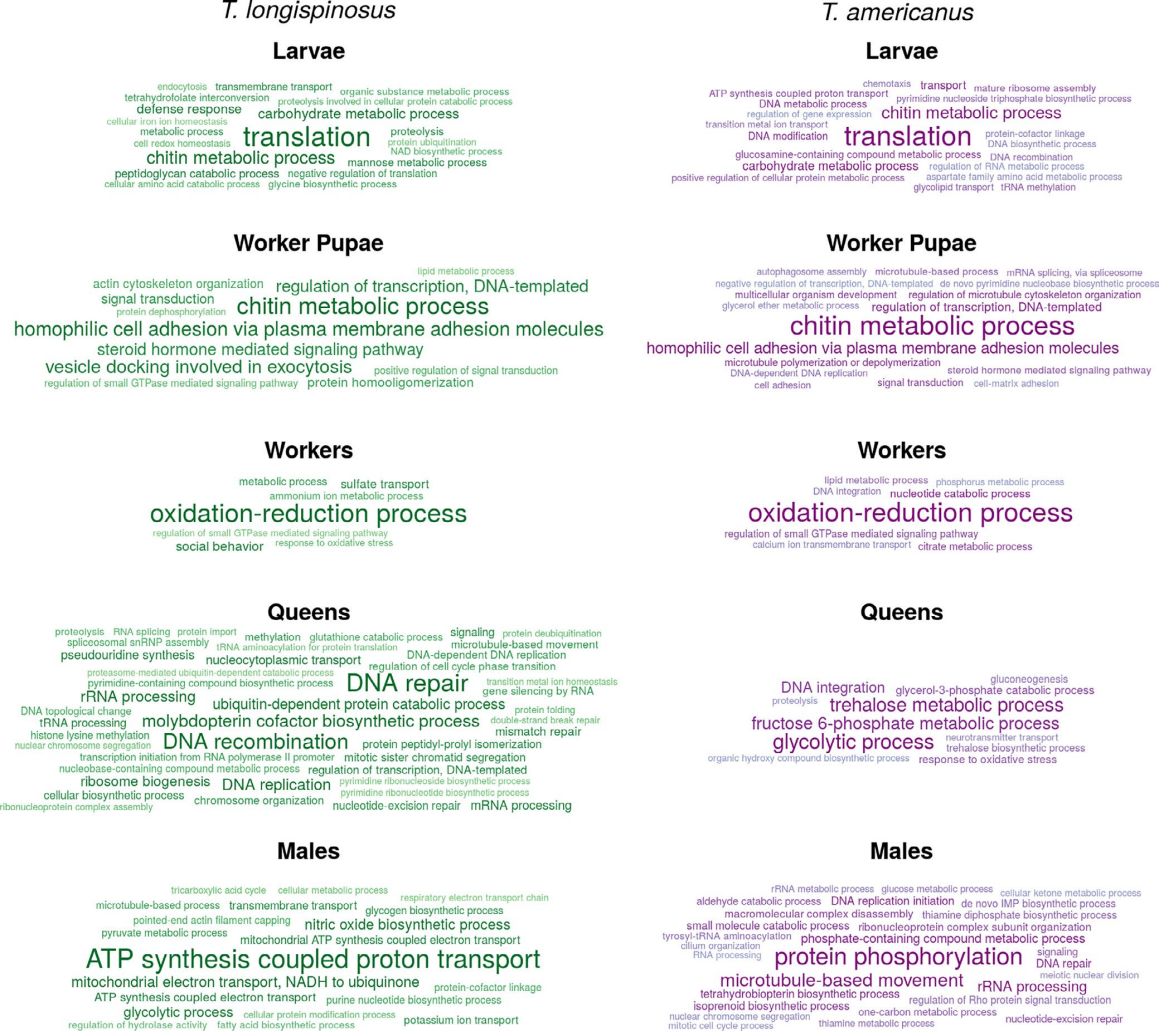
Results of the GO enrichment analyses based on genes uniquely overexpressed in each of the five groups (larva, worker pupa, worker, queen, and male) of the slave‐making ant *Temnothorax americanus* and its main host *Temnothorax longispinosus*. GO enrichment was calculated using topGO using the weight01 algorithm and comparing GO annotations of biological processes of the lists of uniquely overexpressed genes with the ones of the whole transcriptome using Fisher's exact test. Displayed are only terms with a *p*‐value below .05; font size negatively correlates with *p*‐values

GO enrichment analysis revealed that genes of the same functionality were differentially expressed in larvae, worker pupae, and workers of the two species (Figure 2). In larvae, this included genes with the functions “translation” and “chitin and carbohydrate metabolism,” which are both indicative of growth. The most outstanding gene function in worker pupae of both species was “chitin metabolic process,” pointing to the production of the cuticle as a major process during the pupal stage. In addition, genes with “cell adhesion functions” were upregulated in pupae of both species. Differentially expressed genes in workers of the two species were dominated by the function “oxidation‐reduction process.” Workers are more active than the other groups and this possibly explains this interspecies commonality in gene expression in workers. We found the enriched term “social behavior,” based on one gene of *Gp9‐like* pheromone binding proteins, only among the DEGs of workers of the host *T. longispinosus*.

The two most prominent GO terms in *T. longispinosus* queens*,* “DNA recombination” and “DNA repair,” might be associated with their longevity and fecundity. Similarly, *T. americanus* queens upregulated genes linked to trehalose metabolic processes and other sugar‐related catabolic and metabolic processes. *Temnothorax americanus* queens also upregulated genes linked to response to oxidative stress, because either they suffer from increased oxidation or, more likely, prevent damage from oxidative stress, which also plays a role in aging (Finkel & Holbrook, 2000). Both queens and to a lesser extent workers of *T. americanus* upregulated genes (21 genes in total) with the functionality “DNA integration,” which is strongly associated with the activity of transposable elements.

Predominant GO terms in *T. longispinosus* males were “ATP synthesis‐coupled proton transport” and diverse “biosynthetic processes,” while GO terms in *T. americanus* males included “protein phosphorylation” and “intracellular transport.” Interestingly, neither queens nor males shared many genes and gene functions across species, indicating species‐specific gene expression in reproductives.

### 3.4 Analyses of orthologs

**Figure 3:**
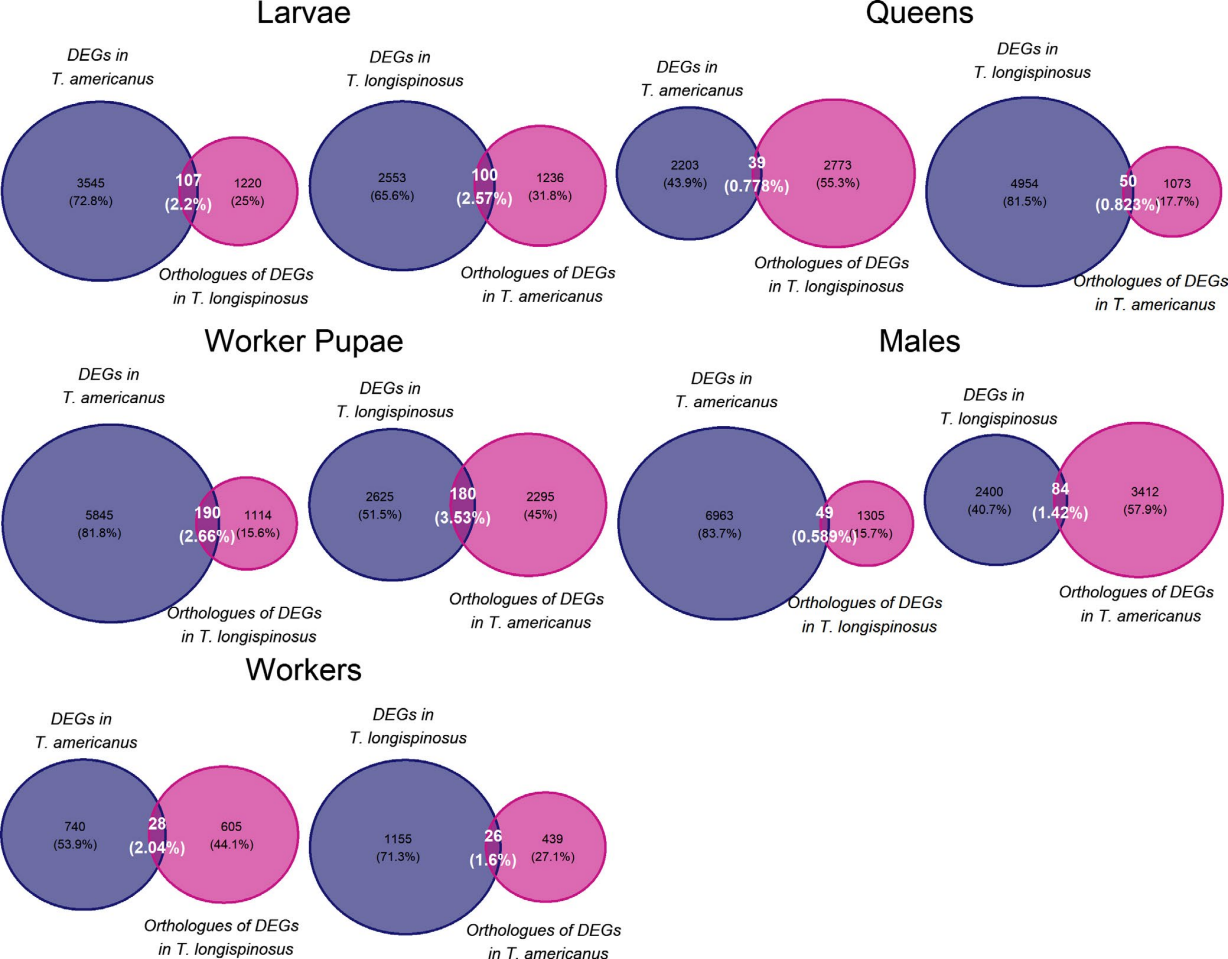
Overlap of overexpressed genes across species. Orthologous genes of uniquely overexpressed genes were searched for in the respective other species, and these were compared to the actual uniquely overexpressed genes in this other species. The figures on the left show the number of unique DEGs found in one phenotype in the slavemaker *Temnothorax americanus* (in purple), and the orthologs of the unique DEGs of its host, *Temnothorax longispinosus* and *T. americanus* in pink. The intersections are those genes found differentially expressed in both species of the respective group. The figures on the right show the unique DEGs of *T. longispinosus* in pink and the respective overlap of orthologous genes of unique DEGs in *T. americanus*

We first tested whether the same genes were responsible for group‐specific gene expression in both species by searching for an overlap between the differentially expressed genes of one species and the orthologs of these differentially expressed genes in the other. With less than 5%, this overlap was generally small, but it was higher in the pupal and larval stages (mean 2.7%) than in the adult stages (1.2%; Figure 3).

**Figure 4:**
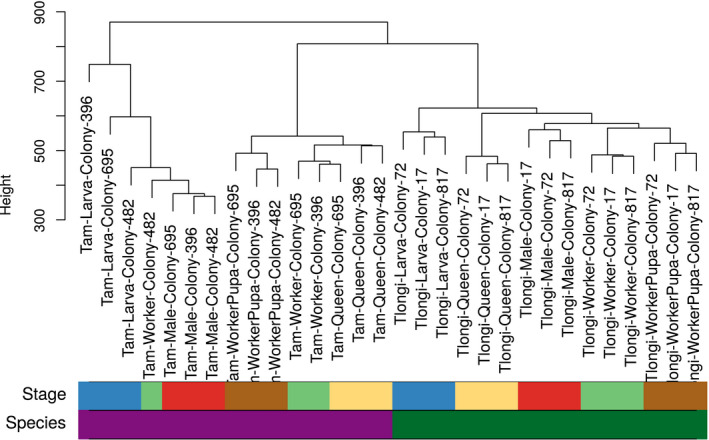
Dendrogram of samples of the slave‐making ant *Temnothorax americanus* and its main host *Temnothorax longispinosus* based on single‐copy orthologous genes only. Clustering was performed using UPGMA of Euclidean distances between orthologous genes of samples of both species

Our orthology analyses revealed 30,758 orthologous genes in the two species, on which we based the following comparisons. A dendrogram of the samples of the two species based on these orthologs revealed three clusters, the outgroup being the larvae, males, and one odd worker of *T. americanus* (Figure 4). Samples of the same developmental stage, caste, or sex never clustered with representatives of the other species, but always with members of their own species. The topology of the dendrogram within species resembled that of the species‐specific analyses (Figure 1a,c).

For single‐copy orthologous genes upregulated in the host *T. longispinosus* compared to the slave‐making ant *T. americanus* and vice versa, we found the smallest difference in the number of DEG between *T. longispinosus* and *T. americanus* workers (245, 0.8%). Indeed, only 76 orthologous genes were found to be upregulated in the host *T. longispinosus*, in which workers take over the normal worker tasks, compared to *T. americanus*, in which workers mainly focus on raiding and reproduction. More differences in gene expression were found between *T. longispinosus* and *T. americanus* queens, which differentially expressed 1,916 genes (6.23%), and especially between males, for which we found the strongest transcriptomic differences (3,103 genes, 10.09%).

**Figure 5:**
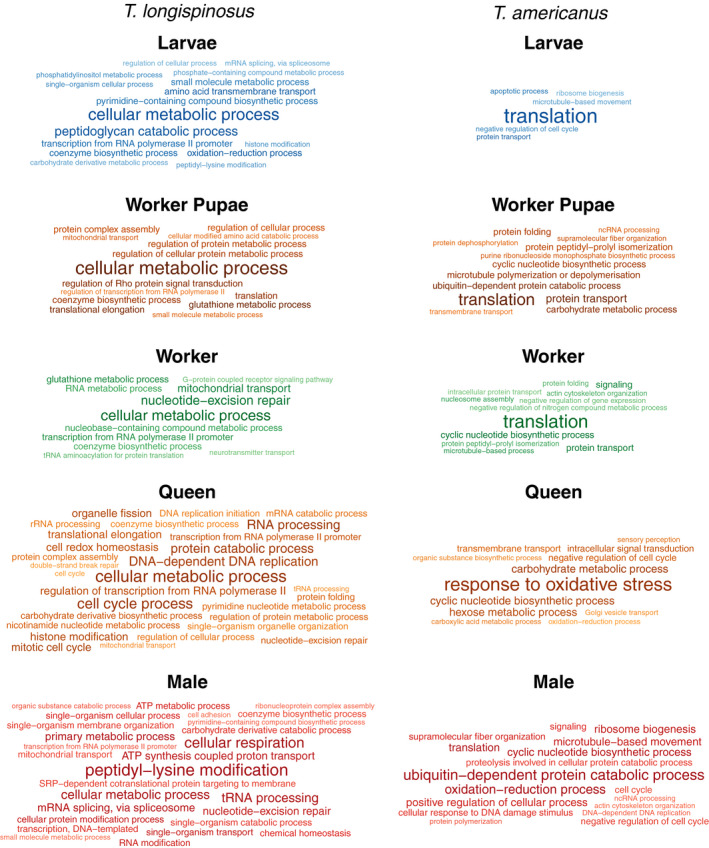
Results of the GO enrichment analyses based on orthologous genes overexpressed between the slave‐making ant *Temnothorax americanus* and its main host *Temnothorax longispinosus* in the five groups (larva, worker pupa, worker, queen, and male). Gene lists were created using only the single‐copy orthologous genes between *T. americanus* and *T. longispinosus* as input for DESeq2, using phenotype and species as well as their interaction as explanatory variables and only taking genes with an adjusted *p*‐value below .05. Displayed are only terms with a *p*‐value below .05. Font size negatively correlates with *p*‐values

The GO enrichment analysis based on differentially expressed genes between species revealed that all castes, sexes, and developmental stages of *T. longispinosus* upregulated genes with “cellular metabolic process” functionality compared to the same stages in *T. americanus* (Figure 5). In *T. americanus,* conversely, we found that all groups but the queens upregulated genes linked to “translation.”

Whereas the genes upregulated in *T. longispinosus* queens involved cellular metabolism, DNA repair and RNA processing functionalities, those upregulated in *T. americanus* were largely linked to “response to oxidative stress.” Similarly, sensory processing genes were upregulated in slavemaker queens. In addition to an upregulation of “cellular metabolic process” genes, host workers showed an upregulation of genes associated with anti‐aging or body maintenance functionalities, such as “nucleotide excision repair” and “glutathione metabolic process.” *T. americanus* workers instead upregulated more genes involved in translation, protein folding, and transport, which might be indicative of *T. americanus* workers being reproductively active.

## 4 DISCUSSION

### 4.1 Transcriptomic characteristics of developmental stages, castes, and sexes

The transcriptomes of the different developmental stages, castes, and sexes were characterized by gene functionalities important for their respective life phase and/or style. Whereas genes upregulated in larvae and pupae were mainly involved in growth and tissue buildup (e.g., chitin metabolism), those of workers reflected their physical activities (e.g., oxidation‐reduction process). Furthermore, enriched functions in queens highlighted the importance of a high fecundity and a long life. For example, the dominant GO term in *T. longispinosus* queens*,* DNA repair, is associated with anti‐aging strategies (Hart, D'Ambrosio, Ng, & Modak, 1979; Lucas, Privman, & Keller, 2016). Similarly, in *T. americanus* queen transcriptomes were enriched for known longevity pathways, but interestingly different ones. For example, the 2nd most enriched functionality, “trehalose metabolic processes,” is involved in the regulation of life span and resistance to stress in the red flour beetle (Xu, Sheng, & Palli, 2013). Trehalose also plays a role in the insulin‐signaling pathway, which affects insect life span (Broughton & Partridge, 2009; Broughton et al., 2005; Tatar et al., 2001).

“DNA integration,” a GO term enriched in the transcriptomes of both *T. americanus* queens and workers, is associated with the activity of transposable elements. Transposable elements might counterbalance the negative effects of reduced genetic diversity by increasing genetic variation (Giraud, Fortini, Levis, Leroux, & Brygoo, 1997; Serrato‐Capuchina & Matute, 2018). For example, they appear to facilitate the adaptation of invasive ant species to novel environments (Schrader et al., 2014). In obligate social parasites, such as *T. americanus*, the effective population size is considerably smaller than in their hosts (Seifert, 2007). Transposable elements might help the slavemaker to catch up in the arms race with their hosts.

In males, which do not engage in colony activities, but focus on mating and reproduction, the enriched terms point to general cellular functions, which at present are difficult to interpret.

### 4.2 Commonalities and differences in gene expression between species

According to the genetic toolkit hypothesis, specific phenotypic traits are typically controlled by a conserved set of genes across species (Toth & Robinson, 2007; Toth et al., 2010). Nevertheless, other studies showed that molecular pathways or functionalities rather than genes are conserved in evolution (Berens, Hunt, & Toth, 2014). Our analyses point to the latter: Even within the more similar early developmental stages, only less than 3% of the genes were commonly overexpressed in *T. americanus* and *T. longispinosus*, even though in larvae and pupae the same dominant functions were enriched. The more pronounced interspecies similarities during development and in workers compared to reproductives suggest a high degree of conserved molecular pathways in these groups. In bumblebees, gene expression was also more conserved during early development, while adult transcriptomes diverged more (Harrison, Hammond, & Mallon, 2015). In both *Temnothorax* species, larval transcriptomes were characterized by an upregulation of translation genes. This was expected, as protein biosynthesis is an important process during larval growth. Larvae and pupae of both species upregulated genes associated with chitin metabolism, reflecting the importance of the formation of the cuticle during development. Pupae also showed an upregulation of genes with cell adhesion functions, which play a role in the development of different cell types and are essential for epithelial tissue development (Harris, 2012; Rio, 1993). Workers of both species upregulated genes linked to oxidation‐reduction processes relative to all other castes and developmental stages, supporting an earlier finding in *T. longispinosus* (Feldmeyer, Elsner, & Foitzik, 2014). In female reproductives of both species, we found strong signals of anti‐aging mechanism, even though different pathways were utilized. Whereas *T. longispinosus* queens mainly prevent DNA degradation, *T. americanus* queens invest in trehalose metabolism. Moreover, the orthology analysis revealed that slavemaker queens also invest more than host queens in genes linked to the response to oxidative stress. Why queens of the two species are using different molecular pathways to reach a long life span will be investigated in future studies.

### 4.3 Species‐specific expression profiles

*Temnothorax americanus* and *T. longispinosus* differ in numerous traits, all of which might be reflected in divergent gene expression patterns. Nevertheless, gene functions that differ between *T. americanus* and *T. longispinosus* across castes might also provide first insights into the evolution and the consequences of the life history of slave‐making ants. *Temnothorax longispinosus* queens and workers upregulated genes linked to cellular metabolic processes. Host ants might have a higher metabolism, which would match the constantly higher activity level of nonparasitic ants. *Temnothorax americanus* upregulated genes linked to translation in all castes except queens. The upregulation of translation genes might reflect the production and maturation of eggs in the ovaries of *T. americanus* workers, which are much more fecund than host workers (Foitzik & Herbers, 2001).

### 4.4 Conclusions

Despite their close relatedness, the two ant species *T. americanus* and *T. longispinosus* show divergent lifestyles (Beibl et al., 2005; Feldmeyer, Elsner, Alleman, & Foitzik, 2017). In agreement with our expectation, our gene expression analyses revealed similarities across the two species in molecular pathway characteristics for larval and pupal transcriptomes. Though adult workers of *T. americanus* and *T. longispinosus* show distinct behaviors and morphologies, their transcriptomes are characterized by similar gene functions. Queens, however, differed strongly in gene expression and functionalities between *T. americanus and T. longispinosus*. Yet they commonly invested in anti‐aging processes, but utilized different molecular pathways. Future studies involving more slavemaker and host species will reveal whether the differences in gene expression between the two species are idiosyncratic or associated with the shift from a free‐living to a socially parasitic lifestyle.

## CONFLICT OF INTEREST

None declared.

## AUTHOR CONTRIBUTIONS

**Claudia Gstöttl:** Conceptualization (lead); data curation (lead); formal analysis (lead); methodology (supporting); writing – original draft (lead). **Marah Stoldt:** Formal analysis (lead); methodology (supporting); visualization (lead); writing – original draft (supporting). **Evelien Jongepier:** Conceptualization (lead); writing – original draft (supporting). **Erich Bornberg‐Bauer:** Conceptualization (lead); Writing – original draft (supporting). **Barbara Feldmeyer:** Formal analysis (supporting); methodology (supporting); writing – original draft (supporting). **Jürgen Heinze:** Conceptualization (lead); writing – original draft (lead). **Suzanne Foitzik:** Conceptualization (lead); writing – original draft (lead).

## Supporting information

Differentially expressed genes from pairwise comparisons between different developmental stages, castes and sexes in *T. americanus*

Differentially expressed genes from pairwise comparisons between different developmental stages, castes and sexes in *T. longispinosus*

Enriched GO terms in uniquely overexpressed genes in different developmental stages, castes and sexes from intraspecific comparison of *T. americanus*

Enriched GO terms in uniquely overexpressed genes in different developmental stages, castes and sexes from intraspecific comparison of *T. longispinosus*

## Data Availability

All raw reads for this study were archived at NCBI's Short Read Archive (SRA) in.fastq format (BioProject ID: PRJNA606685).
